# Acute Onset of Exogenous Endophthalmitis after Dexamethasone Implant Injection Treated without Implant Removal

**DOI:** 10.1155/2018/4614802

**Published:** 2018-11-18

**Authors:** George G. Bastakis, Anastasios Stavrakakis, Avgoustinakis Nikolaos, Dimitris Dimopoulos, George Pappas

**Affiliations:** Ophthalmology Clinic, Medical Retina & Vitreoretinal Surgery Department, Venizeleio Hospital of Crete, Knossos Avenue 44, Heraklion of Crete, Greece

## Abstract

We present a case of acute endophthalmitis after intravitreal dexamethasone implant injection and discuss the management of this rare and challenging case in which the implant could not be removed. A 50-year-old woman with a history of branch retinal vein occlusion in the right eye was treated with intravitreal dexamethasone implant injection for macular oedema. Four days after injection, the patient was admitted to the department with acute pain, decreased vision, and redness. A diagnosis of acute post-intravitreal injection endophthalmitis was made. A 23-guage (23G) vitrectomy was performed immediately to remove the implant, and a vitreous tap for culture and polymerase chain reaction was acquired during the procedure. We were unable to remove the dexamethasone implant during the vitrectomy because of dense membrane formation. At the end of the procedure, we injected intravitreal antibiotics (vancomycin and amikacin), and the patient was treated with fortified topical antibiotics and steroids. At the time of writing, 5 years later, the patient retains a best corrected visual acuity of 10/10 (6/6) with dexamethasone implant therapy maintenance. Intravitreal dexamethasone implant-associated endophthalmitis is a rare and challenging condition. Immediate 23G pars plana vitrectomy, even without removal of the implant, can lead to favourable visual results.

## 1. Introduction

As reported in previous prospective randomized trials, dexamethasone intravitreal implant injection (Ozurdex, Allergan Inc, Irvine, CA) has been shown to be an effective treatment option for a variety of pathological conditions, including diabetic macular oedema (DMO), secondary macular oedema (MO) after retinal vein occlusion (RVO), and noninfectious posterior uveitis [1–3]. Long-lasting potency of the implant has been shown to be able to relieve the burden of monthly treatment with anti-vascular endothelial growth factor (anti-VEGF) in resistant cases of MO secondary to diabetes or RVO. The rate of complications with this treatment is low, and the most common side effects are cataract formation in phakic patients and an increase in intraocular pressure (IOP) [4]. Although cases of post-intravitreal injection endophthalmitis are not infrequent [5], endophthalmitis following dexamethasone implant injection is rare, and few case reports describing this side effect have been published in the literature [6–8]. Because different pharmacological properties can affect infection features, the optimum treatment for post-dexamethasone implant endophthalmitis can differ from the optimum treatments for endophthalmitis from other causes (cataract surgery or intravitreal injection) [9]. In two of the four published cases, the intravitreal implant was removed after vitrectomy, and intravitreal antibiotics (IVABs) were used [6, 7]. In the other two published cases, repeated IVAB injections were used as treatment without vitrectomy or implant removal [8]. To our knowledge, this is the first case report describing endophthalmitis after dexamethasone implant injection managed with 23G vitrectomy without implant removal and followed by administration of IVABs.

## 2. Case Presentation

A 50-year-old Caucasian woman with no previous ocular pathologies was admitted to our department in 2011 presenting with reduced vision and metamorphopsia in her right eye. Her best corrected visual acuity (BCVA) was 2/10 (6/30) in the right eye and 10/10 (6/6) in the left eye. A clinical examination revealed branch retinal vein occlusion (BRVO) in the inferotemporal vein with secondary MO. Over the course of the subsequent 6 months, anti-VEGF (ranibizumab) treatment was administered, resulting in improved visual acuity without complete resolution of the MO. We then opted to treat the patient with dexamethasone intravitreal implant injection. The procedure was performed in the operating room under topical anaesthesia and sterile conditions. Povidone-iodine periocular scrub and 10% solution were applied to the eyelids, followed by 5% solution to the ocular surface for 3 min. The eye was then draped, and a sterile speculum was used to perform the dexamethasone intravitreal implant injection. After implantation, moxifloxacin 0.5% drops were administered four times daily for 1 week. The patient responded well to the dexamethasone implant and showed BCVA improvement to 9/10 (6/7) and MO resolution lasting for >4 months. Six months after implantation, MO was again present, and BCVA had reduced to 6/9.5. Dexamethasone implant was applied for the second time in the same manner as previously described. On the fourth day after implantation, the patient was admitted to our department with acute pain, redness, and vision loss in her right eye. The right BCVA at that point was 1/20 (6/120). Ophthalmic examination revealed conjunctival injection, mild corneal oedema, grade 3+ anterior chamber cells, hypopyon (1 mm), and an IOP of 8 mmHg. A posterior chamber investigation revealed reduced red reflex and vitreous haze that made observation of retinal detail difficult. The implant was located inferiorly, and fibrous membranes were present in the vitreous cavity with attachment to the retinal tissue. A diagnosis of acute endophthalmitis post implantation was made. On the same evening, a 23G pars plana vitrectomy was performed, and a vitreous tap was acquired at the beginning of the procedure for cultures, sensitivity, and polymerase chain reaction (PCR). We planned to remove the dexamethasone implant using a vitrectome and 23G forceps during the vitrectomy, but this was not possible due to dense membrane formation and low visualisation of the retina at the implantation site. For safety reasons, we opted to leave the implant in place, and at the end of the procedure, we injected vancomycin (1 mg/0.1 ml) and amikacin (0.4 mg/0.1 ml) into the vitreous cavity. Postoperatively, the patient received topical treatment with norfloxacin 0.3% and vancomycin (50 mg/ml) fortified antibiotic drops hourly, atropine drops three times daily, and systemic corticosteroids (8 mg methylprednisolone) daily. Vitreous cultures were positive for* Staphylococcus epidermidis*, and PCR was negative for* Streptococcus* spp.,* Haemophilus influenzae*,* Staphylococcus aureus*,* Pseudomonas aeruginosa*,* Neisseria meningitidis*,* Streptococcus pneumoniae*, and* Listeria monocytogenes*. The patient responded well to treatment, with a gradual reduction in inflammation and hypopyon as well as an improved vision at 48 h after surgery. One week after treatment, a tapering regimen of drops and steroids was initiated. Three months later, the patient's BCVA was 3/10 (6/19), and a third dexamethasone implantation was performed 6 months after the onset of endophthalmitis, resulting in a BCVA of 8/10 (6/7.5) after 4 months. At the time of writing, 5 years after the onset of endophthalmitis, the patient's VA is 10/10 (6/6, after phacoemulsification) in the right eye, and she requires a dexamethasone implantation once a year.

## 3. Discussion

Currently, the use of intravitreal medications for many different ocular pathologies is common in everyday clinical practice. Endophthalmitis is the most serious complication after intravitreal injection and can significantly impact final visual acuity. The risk of endophthalmitis after intravitreal injection has been reported to range from 0.03% to 1.4% and varies by study and pharmaceutical agent [10]. It is not uncommon for post-intravitreal injection endophthalmitis to be managed similarly to postoperative endophthalmitis [9, 11]. Typically, a vitreous sample is acquired for cultures and PCR, IVABs are administered, and, in severe cases, pars plana therapeutic vitrectomy is performed [4, 9, 11]. In contrast to endophthalmitis after intravitreal injection, post-dexamethasone implant endophthalmitis is rare [4]. Indeed, no cases of acute endophthalmitis were reported in the GENEVA study, and only one case was reported in the MEAD study [1, 2]. Of the cases in the literature describing endophthalmitis after dexamethasone implant injection, two were treated with vitrectomy and implant removal in combination with IVAB administration [6, 7]. In the two other cases, IVABs were administered and repeated after 3 days, and neither vitrectomy nor implant removal was performed [8]. The removal of the implant after vitrectomy is recommended by some clinicians because it is hypothesised that the implant can act as a “depot” for the infective organism and that dexamethasone can deteriorate the immune defence of the host.

In our case as well as in the two cases reported by Esen et al. [8], the implant maintenance did not result in any additional complications or in a decreased final VA (in all cases, the final VA was better than that at baseline). Further, at 5 years after infection, the VA of the present case is unremarkable when MO is not present. We hypothesise that immediate vitrectomy rapidly reduced the bacterial load in the present case. Moreover, a combined reduction of ocular inflammation related to antibiotics and microorganism products may have occurred due to the action of dexamethasone. The favourable final outcome in this case is likely also related to the cultured bacterium (*S. epidermidis*), which has been associated with improved prognosis in endophthalmitis patients [9, 11, 12]. Postinjection infections can result from contamination with the commensal flora of patients or surgery staff. Coagulase-negative staphylococci (primarily* Staphylococcus epidermidis*) and* Staphylococcus aureus* are found in the conjunctiva and eyelids and on the skin [13–15]. These, along with oral and nasal commensal flora (primarily streptococci), can spread through droplets [13, 16]. Coagulase-negative staphylococci (primarily* Staphylococcus epidermidis*),* S. aureus*, and* streptococci* have been shown to be common pathogens in postinjection and postcataract endophthalmitis [13, 17].

It is possible that the present case of* Staphylococcus epidermidis* endophthalmitis could have been caused by violation in the injection protocol by any of the surgery staff members. Another possible cause could be the use of contact lenses by the patient the same day she received the injection. It is well known that* Staphylococcus epidermidis* and other microorganisms can be found attached to the surfaces of contact lenses if proper cleaning, maintenance, and wear habits are not followed [18–20]. In order to prevent similar events in the future, we have reeducated the related surgery staff on safety protocols that must be followed during intraocular injections. We also reminded the patient that contact lenses should be used correctly and safely and should not be used for at least 3 days after injection.

The favourable anatomical and visual outcomes of the present case suggest that challenging cases of endophthalmitis after dexamethasone injection can be effectively treated with immediate pars plana vitrectomy and IVAB administration even without implant removal.
